# The Endocannabinoid System as Pharmacological Target Derived from Its CNS Role in Energy Homeostasis and Reward. Applications in Eating Disorders and Addiction

**DOI:** 10.3390/ph4081101

**Published:** 2011-08-10

**Authors:** Maria-Paz Viveros, Francisco-Javier Bermúdez-Silva, Ana-Belén Lopez-Rodriguez, Edward J. Wagner

**Affiliations:** 1 Departamento de Fisiología (Fisiología Animal II), Facultad de Biología, Universidad Complutense, Madrid 28040, Spain; E-Mail: lranabelen@gmail.com; 2 Laboratorio de Medicina Regenerativa, Fundación IMABIS, Hospital Carlos Haya, Málaga 29010, Spain; E-Mail: franciscoj.bermudez@fundacionimabis.org; 3 INSERM U862, Avenir group “Energy Balance and Obesity”, Neurocentre Magendie, Bordeaux 33077, France; 4 Department of Basic Medical Sciences, College of Osteopathic Medicine of the Pacific, Western University of Health Sciences, Pomona, CA 91766, USA; E-Mail: ewagner@westernu.edu

**Keywords:** CB1, CB2, FAAH, energy balance, eating disorders, anorexia nervosa, bulimia nervosa, cannabinoid-based therapy, drug-abuse, addiction

## Abstract

The endocannabinoid system (ECS) has been implicated in many physiological functions, including the regulation of appetite, food intake and energy balance, a crucial involvement in brain reward systems and a role in psychophysiological homeostasis (anxiety and stress responses). We first introduce this important regulatory system and chronicle what is known concerning the signal transduction pathways activated upon the binding of endogenous cannabinoid ligands to the G_i/0_-coupled CB1 cannabinoid receptor, as well as its interactions with other hormones and neuromodulators which can modify endocannabinoid signaling in the brain. Anorexia nervosa (AN) and bulimia nervosa (BN) are severe and disabling psychiatric disorders, characterized by profound eating and weight alterations and body image disturbances. Since endocannabinoids modulate eating behavior, it is plausible that endocannabinoid genes may contribute to the biological vulnerability to these diseases. We present and discuss data suggesting an impaired endocannabinoid signaling in these eating disorders, including association of endocannabinoid components gene polymorphisms and altered CB1-receptor expression in AN and BN. Then we discuss recent findings that may provide new avenues for the identification of therapeutic strategies based on the endocannabinod system. In relation with its implications as a reward-related system, the endocannabinoid system is not only a target for cannabis but it also shows interactions with other drugs of abuse. On the other hand, there may be also a possibility to point to the ECS as a potential target for treatment of drug-abuse and addiction. Within this framework we will focus on enzymatic machinery involved in endocannabinoid inactivation (notably fatty acid amide hydrolase or FAAH) as a particularly interesting potential target. Since a deregulated endocannabinoid system may be also related to depression, anxiety and pain symptomatology accompanying drug-withdrawal states, this is an area of relevance to also explore adjuvant treatments for improving these adverse emotional reactions.

## 1. Introduction

### 1.1. Endocannabinoid System

The endocannabinoid system (ECS) is a lipid signaling system which includes the cannabinoid receptors, the endogenous lipid ligands (endocannabinoids), and the enzymatic machinery for their synthesis and inactivation [1,2]. It is a highly conserved system along the phylogeny and its better known and studied function is the modulation of neurotransmission [3-5]. In the central nervous system it is involved in other processes like plasticity and hippocampal neurogenesis [6], neural progenitor cells differentiation [7], psychiatric disorders [8], emotions [9], anxiety, stress [10,11], neuroprotection (reviewed in [12]), antinociception [13] and immunomodulation [14]. This review will highlight what is currently known regarding diverse cannabinoid modulation of signal transduction and synaptic transmission, interactions with hormones and other neuromodulators [15], and the central regulation of energy balance [16], as well as reward and addiction pathways [17,18].

This leads to the idea that modifications in the ECS signaling could be a very useful tool for future treatments of a variety of different diseases and disorders. There are many current studies and clinical trials evaluating cannabinoid compounds for liver disease [19], multiple sclerosis [20], gastric cancer [21] or Huntington disease [22] that underscore the potential therapeutic utility of cannabinoids-like compounds.

### 1.2. Endocannabinoid Compounds

Endocannabinoid compounds are amides, esters and ethers of long-chain polyunsaturated fatty acids that are synthesized on demand [2,23]. N-arachidonoylethanolamine or anandamide (AEA) and 2-arachidonoylglycerol (2-AG) are the most studied endocannabinoids and were discovered first by Devane *et al.* [24], and Mechoulam *et al.* [25] and Sugiura *et al.* [26], respectively. Since then, other endocannabinoids agonists have been identified; including 2-arachydonyl-glyceril-ether (noladin ether) [27]; O-arachydonoyl-ethanolamine (virodhamine) [28]; N-arachidonoyl-dopamine (NADA) [29]; N-oleoylethanolamine (OEA) and palmitoylethanolamide (PEA) [30]. However, OEA and PEA are not strictly considered endocannabinoids because they do not target the cannabinoid receptors.

Due to their lipophilic structure, endocannabinoids cannot be stored in vesicles. Instead they are synthesized and released when needed, “on demand” ([2,3], recently reviewed in [31]). As such they are not considered “classical neurotransmitters” but they play a very important role in synaptic transmission and neuromodulation, particularly in glutamatergic and GABAergic synapses [15]. Termination of endocannabinoid signaling is mediated by cellular reuptake and enzymatic hydrolysis. The reuptake transporter of endocannabinoids has not been indentified yet, there is only pharmacological evidence of its existence through the use of reuptake inhibitors (e.g., AM404; VDM11; OMDM-1; OMDM-2; UCM707) [see 16 for review]. However, the enzymes involved in the metabolism of AEA and 2-AG are better known and described. 2-AG is degraded by the monoacylglycerol lipase (MAGL) giving rise to free fatty acids and glycerol [32]. Fatty acid amide hydrolase (FAAH) is the main AEA hydrolase and it degrades this endocannabinoid to arachidonic acid and ethanolamine in a tissue dependent manner [33]. As we will discuss later, mutations in the FAAH genetic code may result in the dysregulation of endogenous cannabinoid signaling, thereby producing alterations in the myriad regulatory processes with which they are involved, within them, we will focus in FAAH role in addiction.

### 1.3. Cannabinoid Receptors

There are different types of receptors to which cannabinoid compounds can bind. These include the classical cannabinoid receptor subtypes (CB1 and CB2), as well as non-CB1/non-CB2 receptors, the transient receptor potential cation channel subfamily V member 1 (TRPV-1), peroxisome proliferator-activated receptors (PPAR), and the orphan receptors GPR55 and GPR119 (see [34] for review). The activation of TRPV-1 by endocannabinoids results in the modification of intracellular calcium levels [34]. OEA and PEA can bind to PPARα, which then increases the synthesis of neurosteroids [35]. GPR55 is mainly activated by AEA and PEA [36] and its activation also leads to increases in intracellular calcium [37]. Finally, GPR119 binds OEA and PEA, which then activates the adenylate cyclase (AC) leading to increases in the levels of cAMP [38,39].

Table 1 summarizes some of the most recent studies regarding the implications of these receptors in aspects such as energy balance, eating disorders or addiction and drug abuse.

## 2. Signal Transduction

### 2.1. Pathways and Effector Systems Elicited Following Cannabinoid Receptor Activation

Cannabinoid agonists interact primarily with two subtypes of receptors: the CB1 and CB2 receptors. CB1 receptors are expressed in neurons of the central and peripheral nervous systems [52-56] the pituitary gland [57] the gastrointestinal tract [58,59] and the immune system [60,61]. While at first CB2 receptors were believed to be expressed primarily in cells and tissues comprising the immune system [62,63], they have since been localized in the brainstem and mediobasal hypothalamus [64,65]. Both receptor subtypes also are found to varying degrees in pre- and post-meiotic male gametes [66], and in the preimplantation embryo [67]. Thus the degree of overlap in the distribution of these receptors is greater than originally thought.

As mentioned briefly above, CB1 and CB2 receptors are metabotropic, G_i/0_-coupled receptors, and when activated by endogenous or exogenous cannabinoids they trigger dissociation of the heterotrimer and a reduction in adenylyl cyclase (AC) activity, cAMP production and protein kinase A (PKA)-dependent phosphorylation [67-71]. This fundamental step in CB1 and CB2 receptor signaling is instrumental for a number of different effector pathways regulated by cannabinoids. At the cellular level, CB1 receptors modulate stimulus-secretion coupling, as well as neuronal excitability through both presynaptic and postsynaptic mechanisms. For example, cannabinoid agonists presynaptically inhibit glutamatergic synaptic currents in hippocampal cultures [72], as well as in many other brain regions including, but not limited to, prefrontal cortex [73] periaqueductal gray [74], striatum [75], cerebellum [76] and mediobasal hypothalamus [77,78]. They also presynaptically inhibit GABAergic synaptic currents in the rostral ventromedial medulla [79], corpus striatum [80], hippocampus [54,55] and substantia nigra [81]. In addition, cannabinoids presynaptically inhibit norephinephrine release from postganglionic sympathetic nerve endings [52]. Moreover, they decrease prolactin release from lactotroph-derived GH4C1 cells [82]. These effects can be attributed to the CB1 receptor-mediated decrease in Ca^2+^ influx through high-threshold (e.g., N-type, P/Q-type, R-type) Ca^2+^ channels as has been documented in AtT20 cells transfected with the CB1 receptor [83], NG108-15 cells [84,85], GH4C1 cells [82], cerebellar Purkinje cells [86] and calyx synapses in the medial nucleus of the trapezoid body [87]. Interestingly, the release of endogenous cannabinoids caused by depolarization of hippocampal pyramidal neurons, cerebellar Purkinje cells, mesolimbic dopamine neurons, serotonergic dorsal raphe neurons and hypothalamic melanin-concentrating hormone neurons can inhibit GABA and/or glutamate release in a retrograde, trans-synaptic manner by phenomena known as the depolarization-induced suppression of inhibition (DSI) [88-92] and excitation (DSE) [92-96] respectively. Moreover, the glucocorticoid-induced suppression of glutamatergic input into the hypothalamic paraventricular nucleus (PVN) is mediated *via* the retrograde actions of endogenous cannabinoids released from parvocellular neurosecretory cells [97-99]. Postsynaptically, cannabinoids augment the A-type K^+^ current (I_A_) in hypothalamic proopiomelanocortin (POMC) neurons and in cultured hippocampal neurons by shifting the inactivation curves to the right in an adenylyl cyclase/cAMP/PKA-dependent manner [100-103]. They also inhibit the M-type K^+^ current in hippocampal CA1 pyramidal neurons [104]. In addition, cannabinoids activate an inwardly-rectifying K^+^ conductance AtT20 cells [83], in oocytes co-expressing the rat brain CB1 receptor and the G-protein gated inwardly-rectifying K^+^ channel GIRK1 [105], and in POMC neurons [78]. Collectively, these findings suggest that cannabinoids decrease stimulus-secretion coupling, and electrochemical transmission between neurons *via* presynaptic, trans-synaptic and postsynaptic mechanisms.

Cannabinoid receptor activation may also elicit a number of other, different signal transduction pathways. For example, the cannabinoid-induced decrease in AMP-dependent protein kinase (AMPK) activity in adipose tissue and liver facilitates lipogenesis and attenuates gluconeogenesis [106]. In addition, Δ^9^-tetrahydrocannabinol (THC) reduces nitric oxide production in macrophages stimulated with lipopolysaccharide or interferon-γ [69]. On the other hand, it elevates intracellular Ca^2+^ levels in splenic T lymphocytes and HPB-ALL cells but not in Jurkat E6-1 cells expressing dysfunctional CB2 receptors [60]. Activation of CB1 and CB2 receptors also induces Erk 1/2 mitogen-activated protein kinase activation in microglia [107,108], whereas activation of CB2 but not CB1 receptors stimulates this pathway in spermatogonia [66]. This vast array of CB1 and CB2 receptor-mediated signal transduction mechanisms undoubtedly contributes to the diverse biological processes influenced by cannabinoids such as learning and memory, antinociception, motor function, energy balance, stress, thermoregulation, reproduction, drug reward and immunomodulation.

### 2.2. Hormonal and Neuromodulatory Influences on the Cannabinoid System

Endogenous cannabinoid biosynthesis and cannabinoid receptor-mediated signal transduction is altered by a number of hormones and neuromodulators, and nowhere is this more apparent than with regard to the cannabinoid regulation of energy homeostasis (see also next section). For example, the expression of CB1 receptors in vagal afferents innervating the stomach and duodenum is increased by fasting and decreased by the satiety hormone cholecystokinin [59]. The anorexigenic hormone leptin decreases hypothalamic concentrations of anandamide (AEA) and 2-arachidonoylglycerol (2-AG), whereas orexigenic ghrelin increases the amount of 2-AG [109,110]. In addition, leptin blocks the glucocorticoid-induced increase in endogenous cannabinoid production in the PVN, whereas ghrelin's hyperphagic effect is ablated in CB1 receptor knockout mice [110,111]. Cannabinoids also regulate POMC gene expression in a sexually differentiated manner [112]. The cannabinoid-induced hyperphagia and hypothermia observed in guinea pigs is sexually disparate; with males being more responsive than females [113]. The latter is associated with differential presynaptic inhibition of GABAergic input onto POMC neurons [113], as well as discrepant activation of GIRK and the I_A_ in these cells [78,102]. These observations are in keeping with sex differences observed in β-endorphin knockout mice, in which males exhibit a more pronounced hyperphagia and resultant obesity with *ad libitum* access to food, as compared to their female counterparts [114,115]. Estrogens further diminish sensitivity to the hyperphagic and hypothermic effects of cannabinoids in females, which can be attributed, at least in part, to the rapid and sustained attenuation of the cannabinoid-induced presynaptic inhibition of glutamatergic input onto POMC neurons, as well as the cannabinoid-induced activation of the I_A_ [103,116]. Thus, the regulation of energy balance is replete with examples of how hormones and neuromodulators can “fine-tune” cannabinoid input onto this system.

## 3. The Endocannabinoid System in Energy Homeostasis and Reward and Addiction Pathways

### 3.1. Endocannabinoid System and Energy Balance

The endocannabinoid system (ECS) is critically involved in the central and peripheral regulation of energy balance. It is strategically located in all the key points involved in food intake and energy expenditure. Thus, it is perhaps one of the few that can coordinate all the players involved in energy balance [1,117].

The peripheral ECS regulates energy balance mainly by helping to control the lipid and carbohydrate metabolism and also it seems to modulate the secretion of important hormones such as insulin, glucagon and adiponectin (reviewed in [16,118]). Endocannabinoids and CB1 receptors are expressed in the gut, the liver, white adipose tissue, skeletal muscle and pancreas, and as such the ECS is poised to play an integral role in regulating gut synthesis of orexigenic and anorexigenic factors as well as gastrointestinal motility and secretion, lipid metabolism and glucose homeostasis (reviewed in [16]). Whereas the functional relevance of the ECS in gastrointestinal tract, skeletal muscle and pancreas still deserves more investigation, its role in the adipose tissue and liver is better established. The ECS in adipose tissue regulate not only the enzymes involved in lipolysis and lipogenesis [119,120], but also adipocyte differentiation from preadipocytes [121] and even transdifferentiation of adipocytes to mitochondria-enriched brown adipocytes [122,123]. In the liver, CB1 signaling promotes lipid synthesis and, especially under high fat diet, the development of liver steatosis, hyperglycemia, dyslipidemia and insulin resistance [124,125].

Centrally the ECS appears to control food intake *via* complex interactions between the hypothalamus, limbic system [138] and brain stem. The hypothalamus is a key brain structure in energy homeostasis because of its ability to integrate signals from central and peripheral tissues and cells. The hypothalamic feeding circuitry consists of orexigenic elements in the lateral hypothalamic area, which harbors orexin and melanin-concentrating hormone (MCH) somata [126,127], and in the arcuate nucleus where neuropeptide Y (NPY)-/agouti-related peptide (AgRP)- and ghrelin-containing neurons are located [126,128,129]. On the other hand, the ventromedial nucleus functions as a prominent satiety center [130,131], and the arcuate nucleus houses the anorexigenic proopiomelanocortin (POMC) neurons that express the posttranslational byproducts α-melanocyte stimulating hormone (α-MSH) and β-endorphin, as well as cocaine- and amphetamine-regulated transcript (CART) [114,132,133]. The latter is also found within neurons in the lateral hypothalamic area, as well as in the dorsomedial and paraventricular nuclei [120]. In addition, anorexigenic corticotrophin-releasing hormone (CRH) neurons in the paraventricular nucleus receive afferent signals from the other components of this neuronal feeding network, and therefore serve as a point of convergence and synaptic integration in formulating an efferent response [133]. Many of these neurons respond directly to circulating nutrients such as glucose, fatty acids and amino acids, and hormones such as insulin, leptin, adiponectin, ghrelin, cholecystokinin, peptide YY, and adrenal and gonadal steroids [111,129,134-138]. Finally, these humoral factors influence cell excitability by signaling through a variety of different enzymatic cascades including, but not limited to, phospholipase C (PLC), protein kinase C (PKC), protein kinase A (PKA), phosphatidylinositol-3-kinase (PI3K), AMP-dependent protein kinase (AMPK), neuronal nitric oxide synthase (nNOS), the mammalian target of rapamycin (mTOR), and the JAK/STAT pathway [111,136-140]. These hormonal and nutrient mediators, along with their signal transduction pathways, effects on neuronal activity and gene transcription as well as energy balance, are highlighted in Table 2.

CB1 receptors are distributed in the nuclei comprising the hypothalamic feeding circuitry, and they modulate the release and expression of orexigenic or anorexigenic signals [1]. Cannabinoid agonists focally administered into the ventromedial nucleus stimulate energy intake [141]. Endogenous cannabinoids also act in the lateral hypothalamic area, where they decrease GABAergic input onto melanin concentrating hormone neurons *via* DSI [91]. While cannabinoids have been reported to increase the hypothalamic release of NPY [142], CB1 receptor antagonism reduces food intake in NPY-deficient mice with the same efficacy as it does in wild-type controls [109]; suggesting that NPY neurons do not play a vital role in the cannabinoid regulation of appetite [1]. And although anorexigenic POMC neurons do not appear to contribute to the appetite-modulating properties of cannabinoids in agouti-related peptide over-expressing A^y^ mice [143], cannabinoids do presynaptically inhibit convergent glutamatergic and GABAergic input onto enhanced green fluorescent protein (eGFP)-expressing POMC neurons [77]. A very important signal in the hypothalamus is leptin, an anorexigenic adipokine whose levels are related to the amount of fat stored. Leptin inhibits endocannabinoid production in the hypothalamus [109], thus decreasing food intake. Conversely, hypothalamic endocannabinoids are increased in genetically obese rodents lacking leptin or its receptor, and treatment of these mice with a CB1 antagonist attenuates the hyperphagia [109]. Also, the orexigenic action of glucocorticoids within the paraventricular nucleus of the hypothalamus is modulated through retrograde CB1-dependent signaling [144].

The limbic system is also involved in energy balance because it controls motivated behaviors (see also next section). There is increasing evidence supporting an interaction between mesolimbic reward pathways and the endocannabinoid system to control food intake [145]. It is known that CB1 receptors are expressed in presynaptic glutamatergic and GABAergic nerve terminals in the ventral tegmental area and endocannabinoids are synthesized by ventral tegmental area dopamine neurons, having a role in the fine-tuned regulation of these cells [146]. And while it is still unclear exactly what cell populations express CB1 receptors in the nucleus accumbens (NAc), it seems that endocannabinoids within this area are capable of increasing food intake in a CB1-dependent manner [147]. Additional studies have also reported that endocannabinoids acting in the NAc modulate the palatability of food [148]. Interestingly, the NAc shell sends projections directly to the lateral hypothalamus (LH), thus suggesting its relationship with a hypothalamic area regulating food intake [149]. This relationship could be functionally mediated by the ECS. In fact, anandamide administration into the NAc shell activates several hypothalamic nuclei, including the LH, the dorsomedial nucleus and the paraventricular nucleus (PVN) of the hypothalamus [150]. Another possibility is that the increase in endocannabinoids within the NAc shell might enhance pleasure from food. These observations suggest that food activates common neuronal circuits that are also involved in other motivated behaviors like sex and abuse of drugs (see next section). In the brainstem, another brain area involved in food intake, cannabinoids inhibit synaptic transmission in the dorsal vagal complex and rostral ventrolateral medulla, and decrease the firing of glucose-sensitive neurons in the nucleus of the tractus solitarius [79,151,152].

### 3.2. Endocannabinoid System and Reward and Addiction Pathways

The reward system is a group of brain structures which regulate and control behaviour by inducing pleasurable effects. It is composed of the ventral tegmental area (VTA), medial forebrain bundle (MFB), NAc, ventral pallidum (VP) and prefrontal cortex (PFC). The major rewarding pathway in the brain is the mesolimbic pathway and goes from the VTA *via* the medial forebrain bundle to NAc, which is the primary release site for the neurotransmitter dopamine. Dopamine is considered the main brain's pleasure chemical and acts on D1 or D2 receptors to either stimulate (D1) or inhibit (D2) the production of cAMP. Among these structures, the NAc and VTA play a critical role in addiction because the NAc (along with the amygdala and PFC) is involved in the establishment of reward-associated memories in humans [183] and the VTA contains the perikarya of the mesolimbic dopaminergic neurons (see [184] for review). They are also involved in the negative experiences of drug withdrawal and abstinence (see [146] for review).

Drug addiction is a chronic brain disorder characterized by complex behavioural and neurobiological changes and processes that leads to drug seeking and loss of control over drug consumption [146]. Addiction is the progression from impulsive to compulsive behaviour of drug taking and it occurs in three stages: (1) binge/intoxication; (2) withdrawal/negative affect; and (3) preoccupation/anticipation. It is thought that these stages feed-back positively with each other, worsening the situation and leading to the final pathologic state [185]. All the drugs of abuse produce pleasurable, learning and reinforcing effects at the first stages (acute use), followed by the neurobiological changes that lead to dependence, addiction and sensitization during the chronic abuse phase, and finally the neuroplasticity phenomenon that give rise to physiological symptoms and craving through the withdrawal state. However, there are also other components including the negative effects of drug abstinence and withdrawal underlying the maintenance of addiction (see [146] for review). Drugs bind to membrane receptors to produce the different physiological and behavioural effects; however, all drugs of abuse share certain processes, brain circuits and neurobiological mechanisms after acute and chronic use [146,186,187].

ECS contributes to the regulation of “normophysiologic” reward [183] that includes sexual activity, nursing, social interactions, play activity and hedonic food intake and, interestingly, these incentive behaviours activate the same brain reward pathways as drugs of abuse [18]. For many years, the dopaminergic pathways and the opioid pathways have been considered as the main systems in mediating reward aspects [187]; however the use of pharmacological or genetic blockage of CB1 receptors and the measurement of the levels of AEA and 2-AG after the activation of reward processes in some recent studies ([187] for review), support the involvement of ECS in reward pathways and drug addiction (see [146] for review).

The ECS regulates motivation and incentive behaviours through interactions with the mesolimbic pathways and it appears that the control of this reward tone is mainly mediated by CB1 receptors. These receptors are present on axon terminals in reward structures and modulate inhibitory and excitatory postsynaptic currents impinging upon mesolimbic dopaminergic neurons (see [18,146,187]). The presence of CB1 receptors in other structures related to reward pathways like the basolateral amygdala, hippocampus (related to memories), hypothalamus (involved in food intake) and cingulate cortex (implicated in learning and cognition) also contributes to this function of the ECS (Maldonado 2006). These receptors also modify the firing rate of the GABAergic neurons [18] and glutamatergic neurons [188] in the NAc, prelimbic cortex and hippocampus, thereby promoting long-term depression (LTD) and long-term potentiation (LTP) [146], drug-related memories and memory-related plasticity phenomenon [189]. In particular, CB1 receptors seem to play a fundamental role in mediating reinstatement of previously extinguished drug-seeking behavior upon re-exposure to the drug or drug-associated cues [189]

As it is already described, the CB1 receptor is critical for the persistence of the psychostimulant substances addiction [189]. Its main ligand is AEA and the key enzyme for AEA metabolism is the FAAH. Indeed, there are many studies showing that this enzyme is highly involved in the mechanism of action and reward pathways activated by different drugs of abuse like alcohol [190], cocaine [191], nicotine [192] or amphetamine [193]. This suggests that the ECS can be strategically targeted in the treatment of drug-abuse and addiction as we will discuss below concerning the role of FAAH.

## 4. Potential of ECS-Based Drugs in Eating Disorders

### 4.1. Pathophysiology of Eating Disorders

Food intake or eating is the process by which edible substances are consumed in order to balance the energy expenditure in living creatures. This process relies in physiologic mechanisms regulating appetite and the natural drive to eat. In some conditions human feeding behavior is altered leading to diseases, collectively known as eating disorders. These are a group of disorders characterized by physiological and psychological disturbances in appetite or food intake. They can be divided into three main pathologies, *i.e.*, binge-eating, Bulimia Nervosa (BN) and Anorexia Nervosa (AN). Binge-Eating Disorder (BED) is associated with three or more of the following: eating until feeling uncomfortably full; eating large amounts of food when not physically hungry; eating much more rapidly than normal; eating alone due to embarrassment; feeling of disgust, depression, or guilt after overeating. Criteria includes occurrence on average, at least 2 days a week for 6 months. The binge eating is not associated with compensatory behavior (*i.e.*, purging, excessive exercise, *etc*.) and does not co-occur exclusively with BN or AN (from DSM-IV, 1994) [194]. BN is characterized by a cycle of binge eating followed by purging to avert weight gain. Purging methods often include self-induced vomiting, use of laxatives or diuretics, excessive exercise, and fasting. AN is characterized by the loss of appetite and is associated with other features including an excessive fear of becoming overweight, body image disturbances, significant weight loss, refusal to maintain minimal normal weight, excessive exercise and amenorrhea [195]. Indeed, AN is classified into two subtypes depending on the presence (AN-BP) or absence (AN-R) of binge-purge behavior and BN is similarly classified into two subtypes depending on the presence (BN-P) or absence (BN-NP) of habitual purging.

In this review we will address the role of the endocannabinoid system (ECS) in eating disorders. We will not include obesity in this review because indeed it is not formally considered an eating disorder. However, we would like to underline the increasing evidence dealing with specific changes in the central nervous system of obese people, including those occurring in brain areas involved in the rewarding aspects of food (reviewed in [196]). Likewise, and maybe reflecting direct central consequences of obesity, it is noteworthy the high incidence of anxiety and depression (also present in classical eating disorders) in obese people, affecting around 50% of this population. Also deserving greater consideration are the striking similarities in the pathophysiologic sequelae occurring with obesity and addiction, also suggesting for a re-evaluation of how these diseases are classified [196].

Eating disorders can be chronic and disabling conditions characterized by aberrant patterns of feeding behavior and weight regulation, including abnormal attitudes and perceptions toward body weight and shape [197]. Indeed, AN has the highest mortality rate among psychiatric diseases [198]. The etiologies of these diseases are at present poorly understood, but both AN and BN occur most frequently in adolescent females. This increased incidence and prevalence may very well be a direct reflection of cultural pressures for thinness [199]. However, the discrete occurrence and heritability suggest there are some biological vulnerabilities involved in these diseases [197]. In fact, twin studies on AN and BN suggest there is a 50–80% genetic contribution to these diseases [200,201]. However, there is little knowledge about the connection between psychological symptoms and the neuropathophysiology associated with these diseases and on how such genetic vulnerabilities impact on brain pathways and what systems are primarily involved. Because of the psychiatric nature of these diseases, the monoamine systems (*i.e.*, the serotonin, dopamine and norepinephrine pathways) have been explored in greater detail. Among these, the serotoninergic system maybe the more adversely affected and its deregulation is present in AN patients. However, the response to selective serotonin reuptake inhibitors is variable among patients suffering different subtypes of the illness, and the efficacy of such medication has been also questioned due to the common occurrence of relapse [202,203].

Current research on eating disorders also points to a deregulation of neuronal circuits involved in food intake, including those related to emotional and reward pathways linked to feeding behavior [204]. In fact, not only neurotransmitters but also neuropeptides and peripheral peptides have been reported to be deregulated in eating disorders patients [reviewed in 205]. Although the abnormal peptide levels could actually be secondary to eating disorders appearance, it has been hypothesized that they could participate in the maintenance and outcome of the disease [205]. Of particular interest for this review, given the tight connection with the ECS, is the deranged leptin signaling system that has been found in AN and BN patients [206,207]. It has been hypothesized that the reward systems could be compromised leading to food intake-related dysphoria that would promote a vicious cycle of decreasing eating in order to avoid the dysphoric consequences of food consumption [197]. Alternatively or additionally, the rewarding aspects of the aberrant eating behaviors might be abnormally increased, mediating in part the patient's addiction to self-starvation [205]. Thus, AN and BN could be considered as dependency syndromes. In this context, the reward system (see Section 3.2) could have an important role since it integrates “liking” (pleasure/palatability) and “wanting” (appetite/incentive motivation) perceptions associated with food.

### 4.2. The Endocannabinoid System in Eating Disorders

The widespread role of the ECS in regulating energy balance (see Section 3.1) has spawned investigations into putative defects in endocannabinoid signaling that may underlie eating disorders. Paradoxically, to the best of our knowledge, there are no animal studies investigating alterations in the ECS in eating disorders, maybe as a consequence of the scarce number of reliable animal models mimicking these diseases. Only a couple of recent studies in the activity-based anorexia paradigm have been performed, and they are focused on the potential therapeutic value of cannabinoids (see next section). Increased blood levels of the endocannabinoid anandamide have been found in both AN and binge-eating disorder (BED) patients, but not in BN patients [208]. Indeed, anandamide levels were significantly and inversely correlated with plasma leptin concentrations in both healthy controls and anorexic women. Interestingly, there is evidence to suggest that hypoleptinemia in AN patients may be an important factor underlying the excessive physical activity [207], one of the hallmarks in AN. Thus, these results suggest that alterations in the ECS associated with deregulated leptin signaling could be involved in the pathophysiology of AN. It is well-known that the ECS and leptin interact functionally at the molecular level (reviewed in [118]), and thus it is easy to draw a theoretical frame in support of the important role played by both systems in AN and the therapeutic potential of leptin and cannabinoids in this disease [204]. Furthermore, elevated levels of CB1 but not CB2 mRNA have been found in the blood of females with AN and BN, further supporting the hypothesis of deregulated endocannabinoid signaling in eating disorders [209]. Paradoxically, these authors found an association between lower CB1 expression and more severe forms of the disorders.

Anandamide belongs to the lipid family of acylethanolamides. Another member of this group of lipids, named oleoylethanolamide, has also an important role on energy balance by promoting satiety and lipolysis through the activation of PPAR-α [210]. This molecule has an anorexigenic action by inducing oxytocin expression in the paraventricular nucleus of the hypothalamus and, interestingly, preliminary clinical results have shown altered levels of OEA in the cerebrospinal fluid and plasma of subjects recovered from eating disorders [48]. These preliminary observations could extend the findings of altered levels of endocannabinoids in eating disorders to a more general involvement of acylethanolamides.

Given the important contribution of genetics to AN and BN (in fact, the heritability estimates are similar to disorders typically viewed as biological like schizophrenia and bipolar disorder) human genetic association studies have been performed in order to identify genes involved in these pathologies, including genes belonging to the ECS. Among these, CNR1 and CNR2 (the genes encoding cannabinoid CB1 and CB2 receptors, respectively), as well as the genes encoding the main enzyme responsible in the degradation of AEA, FAAH. N-acylethanolamine-hydrolyzing acid amidase (NAAA) which functions similar to FAAH but has a different optimal pH, and monoacylglycerol lipase (MAGL) also have been studied. The first family-based study involved fifty two families (parents with one or two affected siblings) that were genotyped for the (AAT) trinucleotide repeat of CNR1 gene. The distribution of alleles transmitted to the patients was not found to be significantly different from the non-transmitted parental alleles. However, upon dividing the samples to restricting and binging/purging subtypes of AN, the data analysis revealed a preferential transmission of different alleles in each of the subtypes, suggesting restricting AN and binging/purging AN may be associated with different alleles of the CNR1 gene [211]. However, a subsequent study involving up to 91 German AN trios (patient with AN and both biological parents) was unable to confirm these results, nor did it show an association for any of 15 single nucleotide polymorphisms representative of regions with restricted haplotype diversity in FAAH, NAAA and MAGL genes [212]. Another study in 115 overweight/obese subjects with BED, 74 non-BED patients with obesity and 110 normal weight healthy controls investigated one of these FAAH polymorphisms, previously implicated in obesity and BED, reporting a lack of association [213] and, in a more recent article, these authors studied the association of this FAAH polymorphism and the CNR1 polymorphism in both AN and BN, in 134 patients with AN, 180 patients with BN and 148 normal weight healthy controls [214]. The authors found a significant increase in the frequency of both polymorphisms in AN and BN patients, a result in sharp contrast with the previous findings by Muller and collaborators that showed a lack of association of these polymorphisms with AN. Additionally, Monteleone and collaborators found a synergistic effect of the two polymorphisms in AN but not in BN. Finally, a couple of recent articles have studied association of CNR2 and GPR55 polymorphisms with anorexia nervosa. The authors detected an association of a CNR2 polymorphism with both AN and BN [44] in a study comprising 204 subjects with eating disorders and 1876 healthy volunteers in Japanese population. Similarly, an association between the GPR55 polymorphism Gly195Val and anorexia nervosa in a study comprising 235 patients and 1244 controls in a female Japanese population [50]. Furthermore, in this latter study the polymorphism functionality was checked in Chinese-Hamster-Ovary cells engineered to express human GPR55. The GPR55 containing the Gly195Val polymorphism was found to induce less phosphorylated extracellular signal-regulated kinase than Gly195 type GPR55 when cells were treated with anandamide. Taken together, the human genetic association studies show evidence of association between ECS genes and eating disorders, but further studies are necessary to definitively confirm these findings.

### 4.3. Therapeutic Use of Cannabinoid Drugs in Eating Disorders

Cannabis preparations have been used for both medicinal and recreational purposes for centuries. Its ancient medicinal use has been primarily related to ameliorate pain and increase appetite in disease states. However, because of their psychostimulant properties and the lack of an adequate body of knowledge, their use in western medicine has been excluded until recently. During the last 20 years this picture has dramatically changed. There has been an exponential increase in the knowledge of the molecular mechanisms underlying cannabinoid effects, and morphological, physiological and pathophysiological studies have shown that the molecular system supporting these effects (*i.e.*, the ECS), is ubiquitous and has a highly relevant role in maintaining whole body homeostasis and, especially, energy homeostasis [1]. This fact has led to an increased interest in the medical use of cannabinoid-related drugs. Thus, in 1985 the Food and Drug Administration approved Marinol (dronabinol) a synthetically-derived Δ^9^-tetrahydrocannabinol (THC) preparation, to relieve nausea and vomiting associated with chemotherapy in cancer patients who have failed to respond adequately to other antiemetics, and in 1992 this compound was also approved for inducing appetite in acquired immune deficiency syndrome (AIDS) patients suffering from cachexia [215,216]. Similarly, nabilone (a synthetic cannabinoid that mimics THC) was also approved in 1985 for ameliorating the nausea of cancer chemotherapy. A more controversial step forward was the use of a cannabinoid CB1 antagonist/inverse agonist (rimonabant) for management of complicated obesity. Although the Food and Drug Administration never approved this drug, the European Medicine Agency did and Acomplia (the commercial name of rimonabant) was in the market for approximately 2 years. Despite the weight loss and improved cardiometabolic profile observed in obese patients, the drug had to be removed from the market due to its undesirable central side effects (reviewed in [16]). More recently, Sativex (the combination of THC and cannabidiol) has been marketed in Canada and European countries like the UK and Spain for the treatment of spasticity due to multiple sclerosis, and it is currently in phase III clinical development for the treatment of cancer pain.

Taken into account the good therapeutic management of cannabinoids in cachexia and malnutrition associated with cancer and AIDS, it looks feasible that this kind of pharmacotherapy could be also useful in the treatment of eating disorders. Unfortunately, there are only two preclinical studies and two small trials assessing cannabinoid treatments in AN. One animal study, performed on the activity-based anorexia paradigm, showed the ability of both Δ^9^THC and the endocannabinoid uptake inhibitor OMDM-2 in increasing food intake, though they were unable to reverse the weight loss in this animal model [217]. By contrast, another recent study using the same animal paradigm have shown that Δ^9^THC is capable of reducing the weight loss associated with the development of AN, *via* a mechanism involving reduced energy expenditure [218]. Regarding the human trials (reviewed in [204]), the former involved 11 AN patients in a four-week crossover trial and THC treatment resulted in increased sleep disturbances and interpersonal sensitivity, whereas there was no significant effect on weight gain [219]. Unfortunately, this study raised several concerns given it was an in-patient study and the occasional tube feeding was used. In addition, THC was compared to diazepam instead of placebo, which could be a confounding factor given diazepam has also been reported to increase food intake per se [220]. The latter involved nine AN out-patients treated with THC. The results showed a significant improvement of depression and perfectionism scores without improving weight gain [221].

Currently, there is an ongoing phase III clinical trial involving 22 subjects to reveal if severe chronic AN patients treated with Marinol have significant improvement on weight, with secondary objectives of the study being evaluation of eating disorder inventory scale, motor and inner restlessness and endocrine parameters (https://www.clinicaltrials register.eu; EudraCT Number: 2007-005631-29). With this very limited number of performed trials (the last one being still not finished) it seems clear that no conclusions can be drawn out regarding the therapeutic validity of a cannabinoid-based approach in eating disorders. However, the satisfactory clinical use of cannabinoid agonists in other pathologies demands and encourages the development of further clinical trials on eating disorders patients.

## 5. Fatty Acid Amide Hydrolase as a Possible Target for Withdrawal Symptoms Treatment: Pharmacological and Genetic Aspects

### 5.1. FAAH Enzymes

Just as CB1 receptor activation is integral to the process of endocannabinoid system (ECS) signaling, so too are the levels of the endogenous ligands and the enzymes that terminate their action. The best known mechanism to end the endocannabinoid signaling is by enzymatic hydrolysis [222]. Here we will focus in the role of the FAAH enzyme that is mainly responsible for the degradation of AEA, controlling its levels to indirectly modulate the CB1 receptor activity that is crucial in regulating reward pathways ([223], reviewed in [18]). There is mounting evidence showing that mutations in FAAH gene may result in the dysregulation of endogenous cannabinoid signaling, thus producing alterations in the various biological processes with which they are involved [190,224]. Despite these advances, the FAAH role in addiction has yet to be explained in depth, although there are some studies and reviews that have clarified some aspects (see[187] for review). In this report, we explore more recent studies and provide updated information about the link between FAAH and addiction.

FAAH is an endoplasmic reticulum localized enzyme that belongs to the family of serine hydrolases [222,225]. They degrade bioactive fatty acid amides like the sleep-inducing substance oleamide and the endogenous cannabinoids AEA and 2-AG [49] to terminate the signaling and thus the function of these molecules. This family of proteins has the amidase signature (AS) that contains a highly conserved 130 amino acid sequence known as the AS sequence. AS enzymes catalyze the hydrolysis of amide bonds (CO-NH2), although this family has evolved and diverged regarding substrate specificity and function [226].

There are two human FAAH enzymes: FAAH-1 and FAAH-2. The FAAH-2 enzyme was identified by Wei and co-workers [225], and it is present in primates, marsupials and other vertebrates, not including mice or rats. The FAAH-2 gene resides on chromosome Xp11.21 and it is 202.4 Kb in length. The gene has three transcripts; two of them are processed transcripts that do not encode a bioactive protein product, with the other transcript containing eleven exons and ultimately translated into the FAAH-2 protein that is 532 amino acids in length. It has many regulatory elements, within them, we can find three microRNA (target prediction): hsa-miR-367; hsa-miR-25 and hsa-miR-363. FAAH-2 gene presents 36 variations producing alterations in essential intronic splice site, giving synonymous coding or modifying the sequence non-coding gene, untranslated regions or introns but it also has some variations affecting coding sequences resulting in amino acid changes with benign phenotype or non demonstrated deleterious sift (www.ensemble.org/Ref:ENST00000374900).

FAAH-1 gene was characterized in 1997 by Gaing and Cravatt [227]. The gene resides on mouse chromosome 4D1 [228] and on human chromosome 1p35-34 [49]. In humans, the gene is 19.5 Kb in length and has seven transcripts, two of which are protein coding. One encodes a peptide fragment 151 amino acids in length that is not well characterized. The other transcript includes fifteen exons [49] and it is translated into the FAAH-1 protein containing 579 amino acids. This gene presents many regulatory elements, within them there are five microRNA (target prediction): hsa-miR-574-5p; hsa-miR-604; hsa-miR-502-5p; hsa-miR-671-5p and hsa-miR-943. FAAH-1 gene has 231 variations resulting in gain of stop codon, frameshift, amino acid changes, silent mutations in coding regions and also some variations in non-coding sequences and untranslated regions (www.ensemble.org/Ref:ENSG00000117480). We will focus on a specific single nucleotide polymorphism (SNP) involved in addiction.

The two enzymes share 20% sequence identity and both are expressed in kidney, liver, lung and prostate. The FAAH-2 enzyme is the most abundant isoform in the heart and ovary, whereas the FAAH-1 enzyme is the most prevalent isoform in the brain, small intestine and testis [225]. There are also some differences in their activity because FAAH-2 hydrolyzes more efficiently the monounsaturated acyl chains (e.g., oleamide) but degrades AEA and PEA with rates only 30–40% those of FAAH-1 [187]. FAAH-1 preferentially hydrolyzes polyunsaturated substrates like AEA, oleamide, palmitic amide and myristic amide to their corresponding acids [187], and also degrades 2-AG to glycerol and arachidonic acid [49].

### 5.2. FAAH-1 Polymorphism and Addiction

The FAAH-1 enzyme has been studied in much more detail. As we said above, this isoform is the most prevalent in the brain, so it probably has a critical role in modulating reward pathways. There are many researchers trying to find a direct link between this gene and drug abuse. However drug addiction is a neurobehavioral disorder of complex origin and the genetic factors are only responsible of the 40–60% of the total risk [229]. Within these genetic factors we can find dopamine receptors [230], nicotinic acetylcholine receptors [231] or histamine receptor [232]. Related to FAAH-1 gene, most of the studies are focused on a single-nucleotide polymorphism (SNP) in the enzyme, a SNP represents a change in a single base pair in a specific position along the genome that may or may not be functionally relevant [49]. In the FAAH-1 enzyme, the SNP means a missense mutation in the amidase domain.

The FAAH-1 SNP (dbSNP rs324420; previously rs57947754), is located in exon three (c.385C>A) and it is linked with addiction and obesity [224,233-235] showing, once more, a relation between these two disorders. It leads to an exchange of proline for threonine at protein position 129 (P129T), and produces a decrease in FAAH expression and net activity [236]. The allele A at rs324420 was found to be associated with street drug abuse and addictive traits [161] in Caucasians and African-Americans, but not in Japanese or other Asian populations [49].

The loss of function produced by the SNP leads to higher endocannabinoids levels that could ameliorate craving and withdrawal symptoms [49] and there is burgeoning evidence showing enhanced anxiolytic and antidepressant effects mediated by AEA [45] that is the main endocannabinoid hydrolyzed by this enzyme. These effects have been tested in pharmacological and genetic FAAH inhibition studies showing that the reduced anxiety behavior is mediated by CB1 receptors [237] that are, as it is explained in Section 3.2, crucial in reward pathways control [18,188].

### 5.3. Studies of FAAH in Addiction and Future Prospects

The studies of FAAH enzyme as a possible treatment for withdrawal symptoms could be divided in pharmacological and genetic studies. Genetic studies are focused mainly in knock out animals and pharmacological studies are pointing to the use of FAAH inhibitors. Table 3 summarizes the more recent pharmacological and genetic studies with FAAH enzyme that are going to be explained in this section.

Mice lacking FAAH enzyme (FAAH-/-) are more responsive to exogenous cannabinoids [49], and they show 15-fold augmented brain levels of anandamide [238]. Due to the well known anxiolytic and antidepressant effects of the endogenous cannabinoid AEA [191], these animals experience less negative effects during abstinence. Some studies prove that FAAH-/- mice show higher alcohol consumption owing to decreased acute ethanol intoxication [239]. The A allele of human SNP produces similar effects to those found in FAAH-/- animals making people nearly five times more likely to drug abuse [49,190,240] because they also have less negative effects during abstinence. Furthermore C/C individuals display more severe withdrawal symptoms and experience more negative effects when they are abstinent than A/A and A/C individuals [240]. Moreover, some studies report that short-interference RNA (siRNA) are able to knockdown the expression of FAAH [241] thus widening the possibilities of genetic manipulation treatments. Another possibility in gene studies is the use of microRNA (miRNA). These are very small RNA molecules able to modulate the expression of many genes, within them, FAAH-1 and FAAH-2 genes have many miRNA as regulatory elements (see Section 5.1 for details). Some groups are now focused in the study of these molecules that control the expression of alcohol-relevant genes [242]. However, gene therapy is at its very first steps and many aspects have to be improved such as sustained gene expression, vector delivery or cell specificity.

The studies with the FAAH inhibitors phenylmethylsulphonyl fluoride (PMSF) or cyclohexylcarbamic acid 3-carbamoyl biphenyl-3-yl ester (URB597) show suppression of cocaine-seeking behavior [191]. URB597 is able to produce higher alcohol consumption because the increased endocannabinoid signaling decreases acute alcohol intoxication [239] and it also enhances the expression of nicotine conditioned place preference [243] because they experience less negative effects when they are abstinent. Moreover, in favor of FAAH inhibition as possible treatment for drug addiction, there are some evidences to suggest that FAAH inhibitors *per se* do not have risk of abuse, do not trigger relapse to drug abuse, and are better tolerated than some CB1 receptor antagonist [192].

Collectively, this leads to the idea of FAAH as a possible target for abstinence treatment by controlling its activity and also its expression. To modulate the activity, several classes of inhibitors have been examined, including reversible (e.g., trifluoromethyl ketones; α-ketoheterocycles) and irreversible (e.g., fluorophosphonates; carbamates; ureas) compounds [244]. As mentioned above, FAAH inhibitors have shown a safer profile than CB1 receptors antagonists like rimonabant, which has psychiatric side effects [192]. Among them, the best known is URB597 but the pharmacological profile of carbamate URB532, α-keto heterocycle OL-135, PSMF, and the most potent of them all, JP104 (which shares carbamate and α-keto heterocycle features) [225], should be evaluated in the framework of addiction.

Another possibility is to control FAAH enzyme expression by modulating its promoter activation. There is some evidence in humans that the FAAH gene promoter is downregulated by estrogen and glucocorticoids by binding to estrogen and glucocorticoid receptors sites independently of the presence of ligands [245], and upregulated by leptin, progesterone [246] and the follicle-stimulating hormone [247]. Thus, the modulation of these hormones could represent another approach to indirectly modify the activity and expression of FAAH in the context of potential treatment for abstinent drug abusers. FAAH inhibition or downregulation should be useful not to avoid addiction but to ameliorate craving, seeking behavior and other withdrawal symptoms.

Further studies are needed to provide more knowledge about the role of FAAH in addiction and also to develop safer inhibitors that exhibit fewer possible side effects, and to improve the delivery and efficacy of siRNA interventions. All of this should be accompanied by social programs to make drug addiction and withdrawal treatments more effective and long-lasting.

## 6. Conclusions

The ECS is a lipid signaling system comprising all the molecular machinery needed to properly activate the cannabinoid receptors. There is a vast array of CB1 and CB2 receptor-mediated signal transduction mechanisms that undoubtedly contributes to the diverse biological processes influenced by cannabinoids. Indeed, endogenous cannabinoid biosynthesis and cannabinoid receptor-mediated signal transduction is altered by a number of hormones and neuromodulators. At the cellular level, cannabinoids decrease stimulus-secretion coupling, and electrochemical transmission between neurons *via* presynaptic, trans-synaptic and postsynaptic mechanisms. At the central nervous system level, cannabinoids are involved in learning and memory, antinociception, motor function, energy balance, stress, thermoregulation, reproduction, drug reward and immunomodulation.

Regarding energy homeostasis, the ECS is strategically located in all the key points involved in food intake and energy expenditure. It is perhaps one of the few that can coordinate all the players involved in energy balance, thus being an interesting target in all the diseases related to an imbalanced energy homeostasis like obesity and eating disorders. The etiology of eating disorders is currently unknown and the molecular systems involved are still largely a mystery. However, an increasing body of evidence points to an important role of brain circuits related with feeding behavior, especially those related with the reward system, where the ECS has an important role. Recent findings, starting from human genetic association studies and including other molecular, physiological and pathophysiological studies, are suggestive of a deregulation of the ECS in eating disorders that is still not completely understood. In clinical practice cannabinoid agonists are being used safely and successfully in other diseases in which weight gain is needed, like cachexia in AIDS patients but, however, the trials on the therapeutic validity of cannabinoids on eating disorders are very scarce and the results are inconclusive. Taken together, all these considerations encourage the development of new clinical trials for assessing cannabinoid agonists in the management of eating disorders.

Intrinsically rewarding stimuli and psychoactive drugs activate the same mesolimbic dopaminergic reward pathway and the CB1 receptor has a key role in the control of this reward pathway. Additionally, the FAAH enzyme indirectly modulates the CB1 receptor activity by ending the cannabinoids action, thus suggesting that it could have a role in regulating reward pathways. A FAAH SNP has been associated with drug addiction and some other evidences suggest a role for this enzyme in drug-abuse. Collectively, the findings have led to the idea of FAAH as a possible target for abstinence treatment by controlling its activity and also its expression. However, further studies are needed to provide more knowledge about the role of FAAH in drug-abuse and addiction and the putative therapeutic value of compounds capable of modulating the activity of this enzyme.

## Figures and Tables

**Table 1:** Recent studies regarding the implication of receptors activated by endocannabinoid compounds, in relevant aspects covered in this review.

**Receptor**	**Involved in**	**Model**	**Brief result**	**Reference**
CB1	Feeding inhibition	Rats	Blockade of CB1 receptor reduces feeding.	40
CB1	Body weight	Rats	Blockade of CB1 receptor reduces food intake which results in long-term reduction of body weight gain.	41
CB1	Smoking cessation	Human	CB1 receptor antagonists may assist with smoking cessation by restoring the balance of the ECS.	42
CB1	Nicotine seeking	Rats	CB1 receptor stimulation increases the reinforcing effects of nicotine and precipitates relapse to nicotine-seeking.	43
CB2	Eating disorders	Mice	There is an association between the R63Q polymorphism of the CNR2 gene and eating disorders.	44
CB2	Food intake	Mice	CB2 knockout mice (-/-) led to greater increases in food intake and body weight with age than in wild type mice.	45
PPAR	Nicotine addiction	Rats	PPAR-α agonists decreased nicotine self-administration and nicotine-induced reinstatement in rats.	46
PPAR	Alcohol seeking	Rats	Activation of PPARγ receptors reduced alcohol drinking and prevented the somatic signs of alcohol withdrawal.	47
PPAR	Eating disorders	Rats	PPAR activated by OEA regulates eating and body weight. OEA levels are altered in the cerebrospinal fluid and plasma of subjects recovered from eating disorders.	48
GPR55	Cannabis use disorders	Gene association studies	CNR1, CB2, FAAH, MGLL, TRPV1 and GPR55 genes have specific influences on cannabis use disorders.	49
GPR55	Anorexia nervosa	Human	The low-functioning Val195 allele of GPR55 appears to be a risk factor for anorexia nervosa.	50
GPR119	Energy balance	Mice/rats	OEA binds to GPR119 to modulate food intake and energy balance.	51

**Table 2:** A summary of the interrelationships between the transduction pathways modulated by hormonal and nutrient signals, and the subsequent effects on excitability, gene transcription and energy balance.

**Hormone/nutrient**	**Transduction pathway**	**Effect on activity/transcription**	**Effect on energy balance**	**References**
Insulin	↑ PI3K & nNOS, ↓ AMPK	↓ POMC, ↑ NPY firing, ↓ NPY expression	anorexigenic	128, 136, 137, 153, 154
Leptin	↑ PI3K, mTOR & JAK/STAT, ↓ nNOS & AMPK	↑ POMC, ↓ NPY	anorexigenic	128, 135, 136-139, 154
Adiponectin	↑ PI3K, AMPK & JAK/STAT	???	orexigenic	155-157
Cholecystokinin	↑ERK/MAPK (in NTS)	↑ POMC (in NTS & diencephalon) & orexin	anorexigenic	158-162
Peptide YY	???	↑↓ POMC, ↓ NPY	Anorexigenic & orexigenic	135, 163-168
Ghrelin	↑ AMPK	↑ NPY, ↓ POMC	orexigenic	110, 135, 169
Glucocorticoids	↑PKA, AMPK	↑ NPY & orexin, ↓ POMC & CRH	orexigenic	110, 111, 170-172
Estrogens	↑PLC, PKC, PKA, PI3K & JAK/STAT	↑ POMC, ↓ NPY	anorexigenic	138, 140, 173-175
Glucose	↓ nNOS & AMPK	↑ POMC & MCH, ↓ NPY & orexin, ↑↓ VMN neurons	anorexigenic	154, 176-180
Amino acids	↑ mTOR	???	anorexigenic	139
Fatty acids	↓ AMPK	↑ POMC, ↓ NPY	anorexigenic	181, 182

**Table 3:** Recent studies regarding the role of FAAH in addiction.

**Type of study**	**Model**	**Brief result**	**Reference**
Genetic	Knockout mice	Specific influences on cannabis use disorders: CNR1, CNR2, FAAH, MGL, TRPV1 and GPR55 genes.	49
Genetic	Knockout mice	FAAH as target for the treatment of neuropsychiatric disorders.	238
Genetic	Knockout mice	Increased endocannabinoid signaling increases ethanol consumption due to decreased ethanol intoxication.	239
Genetic	Human SNPs	Alterations in FAAH activity could explain differences in CB1 function in alcohol dependence.	190
Genetic	Human SNPs	Specific influences on cannabis use disorders: CNR1, CNR2, FAAH, MGL, TRPV1 and GPR55 genes.	49
Genetic	Human SNPs	C385A variance was significantly associated with changes in withdrawal after abstinence.	240
Genetic	siRNA	Small-interfering RNA (siRNA) knockdown FAAH and decreased FAAH protein levels.	241
Genetic	miRNA	microRNA (miRNA) as potential explanation of the polygenic nature of alcoholism.	242
Pharmacological	PSMF/URB597	FAAH inhibitors could be potent modulators of motivational and conditioned aspects.	191
Pharmacological	URB597	Increased endocannabinoid signaling increases ethanol consumption due to decreased ethanol intoxication.	239
Pharmacological	URB597	Increasing endogenous cannabinoid levels magnifies nicotine reward and withdrawal.	243
Pharmacological	URB597	FAAH inhibition present antidepressant and anxiolytic properties.	192
Pharmacological	Several FAAH inhibitors	FAAH enzyme as a target for CNS disorders.	244
Pharmacological	Several FAAH inhibitors	FAAH enzyme isoforms and their inhibitors.	225
Pharmacological/Genetic	Estrogen/Glucocorticoid	Estrogen and glucocorticoid receptors activation modulates transcriptional activity of FAAH gene.	245
Pharmacological/Genetic	Leptin/Progesterone	Leptin and progesterone modulate transcriptional activity of FAAH gene.	246
Pharmacological/Genetic	Follicle-stimulating hormone	Follicle-stimulating hormone (FSH) enhances the activity of FAAH enzyme.	247
